# Description of electroencephalographic data gathered using water-based medium-expansion foam as a depopulation method for nursery pigs

**DOI:** 10.1038/s41598-022-21353-7

**Published:** 2022-10-07

**Authors:** Jack Korenyi-Both, Jorge Vidaurre, Tim Held, Magnus R. Campler, Justin Kieffer, Ting-Yu Cheng, Steven J. Moeller, Andrew S. Bowman, Andréia G. Arruda

**Affiliations:** 1 Department of Veterinary Preventive Medicine, The Ohio State University, Columbus, OH 43215 USA; 2 Pediatric Neurology Department, Nationwide Children’s Hospital, Columbus, OH 43205 USA; 3 Department of Animal Sciences, The Ohio State University, Columbus, OH 43215 USA

**Keywords:** Animal behaviour, Animal physiology, Epidemiology

## Abstract

The United States’ swine industry is under constant threat of foreign animal diseases, which may emerge without warning due to the globalized transportation networks moving people, animals, and products. Therefore, having disease control and elimination protocols in place prior to pathogen introduction is paramount for business continuity and economic recovery. During extraordinary circumstances, it may become necessary to depopulate large populations of animals, including swine, as a disease containment measure. Currently approved depopulation methods for swine present significant logistical challenges when scaled to large populations or performed in field conditions. In the United States, water-based foam is currently approved for poultry depopulation, and recent field studies demonstrate water-based foam is an effective depopulation alternative for swine. While effective, the speed at which water-based foam induces loss of consciousness prior to death, a major welfare consideration, has not been adequately investigated. In this study, 12 nursery pigs were terminated using water-based medium-expansion foam to quantify the time to induce loss of consciousness and ultimately brain death. Each pig was implanted with subdermal electrodes to capture electroencephalographic data, placed in a body sling, and suspended in a plastic bulk container that was subsequently filled with water-based foam. Electroencephalographic data was recorded for 15 min, during which the pigs remained immersed in the water-based foam. Conservatively, average (± SD) time to unconsciousness and brain death was 1 min, 53 s ± 36 s and 3 min, 3 s ± 56 s, respectively. The relatively rapid loss of consciousness compared to other methods limits the amount of distress and is overall a positive finding for the welfare of the pigs that might be depopulated with water-based foam. The findings of this study add additional evidence supporting the use of water-based medium-expansion foam for an emergency depopulation of swine.

## Introduction

The U.S. swine industry is at constant risk of foreign animal disease (FAD) introductions such as African swine fever (ASF) and foot and mouth disease (FMD). The rise of global transportation of feedstuffs, live animals, animal products, and people continually increases the risk for FAD introduction and spread^1–3^. Therefore, in addition to preventing FAD introductions, the U.S. swine industry must prepare contingency plans^4^, including comprehensive depopulation protocols to prevent pathogen spread after an introduction. Depopulation is defined as “the rapid destruction of a population of animals in response to urgent circumstances with as much consideration given to the welfare of the animals as practicable”^5^. Therefore, rapid depopulation of infected swine populations is considered one of the critical actions for containment, control, and elimination of FADs^4^.

The American Veterinary Medical Association^5^ depopulation guidelines outline two classes of depopulation methods in swine: (a) preferred methods; including gunshot, penetrating and non-penetrating captive bolt, electrocution, humane slaughter, inhaled carbon dioxide (CO_2_)_,_ and anesthetic overdose; and (b) methods that are permitted in constrained circumstances; including ventilation shutdown plus heat or CO_2_ (VSD +) and sodium nitrite overdose. Currently approved depopulation methods for swine present significant logistical challenges such as availability of specialized equipment, inability to stockpile CO_2_, and scalability of methods for timely depopulation of large cohorts common in modern swine production. Additionally, equipment leakage and failure can present personnel safety issues with the use of inhalant-based methods such as CO_2_ and N_2_^5–9^. Furthermore, VSD + requires intensive facility preparation, creates a prolonged time interval between implementation and mortality observation^10^, and has been criticized based on swine welfare concerns^5,11^.

In a proof-of-concept study, water-based foam (WBF) was used to rapidly depopulate groups of adult swine after the animals walked out of the housing environment under their own power, thus eliminating the challenge of carcass removal from within buildings^12^. The study showed that a large-scale WBF depopulation field deployment, depopulating batches of 45 cull sows simultaneously, could be carried out safely and reliably with a mean fill time of 1 min, 44 s and a mean cessation of movement latency of 2 min, 8 s post-fill start.

Moreover, WBF can be generated using equipment available in the USDA-maintained National Veterinary Stockpile, which minimizes logistical bottle necks that exist with other methods_._ In addition, the approach presented in Lorbach et al.^12^ is mobile, easy to setup, the foam meets biodegradability requirements^13^, and the process avoids the issue of using lethal gases in the presence of personnel. Another benefit with WBF is the visual obscurement of the depopulation process from personnel performing the task, which may alleviate some of the psychological and emotional stress associated with prolonged participation in depopulation activities^14–16^. Although recent findings indicate WBF is a strong potential candidate for mass depopulation in swine, there is a paucity of information on the effect of WBF on swine physiology and any associated pain or suffering. As with any depopulation method, ensuring rapid loss of consciousness is of utmost importance to minimize swine pain and distress. Electroencephalogram (EEG) has previously been applied to determine time to unconsciousness and isoelectric EEG in swine^17,18^ when stunned by CO_2_, non-penetrative captive bolt^19^, and blunt force trauma^20^, but to our knowledge, has yet to be conducted during WBF depopulation.

The aim of the present study was to establish baseline information on the time to unconsciousness after WBF immersion in nursery pigs using EEG, a critical piece of information necessary to understand the overall utility of the WBF depopulation method.

## Materials and methods

### Ethics and institutional oversight

The animal experiments performed in the present study were approved by The Ohio State University Institutional Animal Care and Use Committee (protocol 2020A00000036). The secondary euthanasia method available on site throughout the trial was a penetrating captive bolt, which would be operated by trained personnel as needed. All pigs were housed and handled according to the Guide for the Care and Use of Agricultural Animals in Research and Teaching. This manuscript was prepared in accordance with ARRIVE guidelines (https://arriveguidelines.org), as applicable for a descriptive study.

### Animal subjects

A total of 12 nursery pigs (male: n = 6; female: n = 6) with an average (± SD) weight of 13.6 ± 1.2 kg (min = 11.7 kg; max = 15.5 kg) were acquired from The Ohio State University Swine Center. All pigs were deemed healthy, had ad libitum access to food and water, and remained in their pen until removed for the trial.

Electroencephalograms (EEG) were performed to quantify the time to unconsciousness and subsequent brain death in pigs subjected to WBF. Given depopulation studies in swine using WBF were unavailable, we based our sample size calculation on a previous study that estimated time to isoelectric EEG using inhaled CO_2_ as the euthanasia method^21^. Assuming a standard deviation of 0.22 (minutes), 12 pigs would allow us to estimate the time to loss of consciousness with a confidence of 95% and a desirable precision of ± 0.125 min^22^.

### Field trial set up and experiment

**Figure 1:**
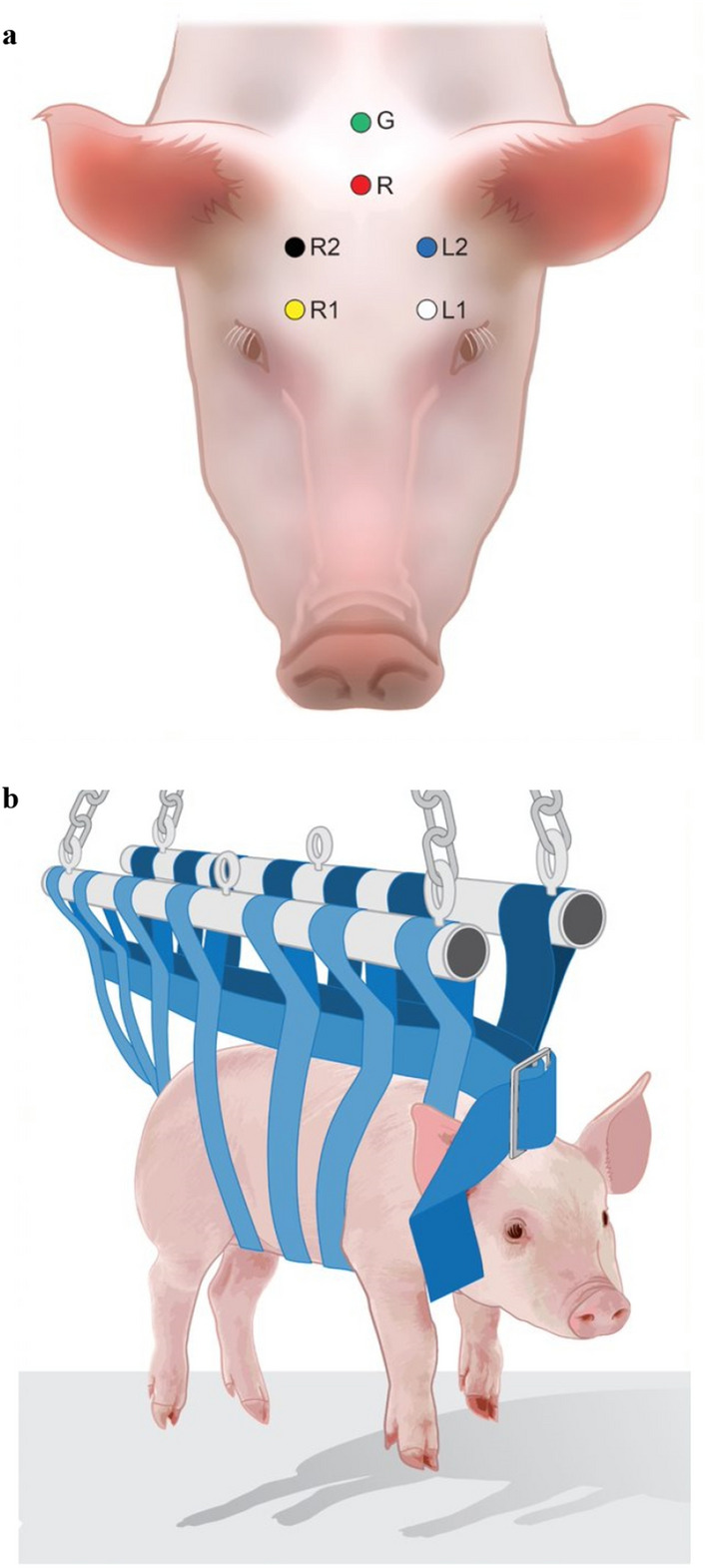
(**a**) Dorsal view of pig showing electrode placement in a six-channel montage (L1, L2, R1, R2, G = ground, R = reference). (**b**) Lateral view of the pig showing orientation of the sling for body support and connections used to suspend the pig within the container throughout the 15 min water-based foaming event. (**a**) and (**b**) were created using Adobe Illustrator v25.4.1 and Adobe Photoshop v22.5.4 (www.adobe.com).

Pigs were individually removed from their holding pens, weighed, and prepped. The rostral portion of the head was shaved and locally anesthetized using 10 ml of lidocaine before six disposable electrodes (120 cm Subdermal Corkscrew Needle Electrodes Ambu, Bellerup, Denmark) were attached based on the montage outlines described by Miller^23^. The electrodes were connected to an EEG transmitter following a four-channel connection (L1, L2, R1, and R2 pattern) with a reference and a ground connection^23^ (Fig. 1). The EEG transmitter (Trackit T4A, Lifelines Neuro, Louisville, KY, USA) was placed into a water-resistant bag to prevent moisture damage and secured on the back of the pig using surgical wrap throughout the foam immersion period.

After the EEG transmitter and electrodes were attached, each pig was placed into a body sling to reduce intensive movements that may interfere with the EEG measurement (Fig. 1). The sling was suspended using a chain attached to a hydraulic-controlled tractor bucket loader and lowered into a plastic bulk container 1.46 m^3^ (1.12 m × 1.12 m × 1.14 m: length, width, and height). The pig remained suspended above the bottom of the container to avoid movements caused by feet gaining traction on the container floor, and to prevent potential dislodging of EEG electrodes by physical contact between the pig and the container.

Foam was applied from the top of the container until the container was filled, and the pig remained fully immersed in the foam during the trial. No top ups of the foam were necessary, and no noticeable breakdown of the foam was observed during the immersion. Following the 15 min dwell time, the individual pig was removed from the foam and death was confirmed by an assessment of respiratory and cardiac arrest, and lack of corneal reflex. Each pig was a replicate, and all pigs were foamed within a 7 h block on the same day to keep equipment, weather, handlers, operators, and data collection as consistent as possible.

### Foam generation

For foam generation, a complete description has been published by Kieffer et al.^24^. In brief, PHOS-CHEK WD881 Class A foam concentrate (Perimeter Solutions, Rancho Cucamonga, CA, USA) was mixed in a 1457 L container with water to create a 1% foam-water solution. A gasoline-powered water pump (AMT Pump Company 2MP13HR, Royersford, PA, USA) was used to deliver the foam-water solution through a medium-expansion aspirated foam nozzle (KR-M4, ANSUL, Marinette, WI, USA).

### EEG collection, data description and interpretation

**Figure 2:**
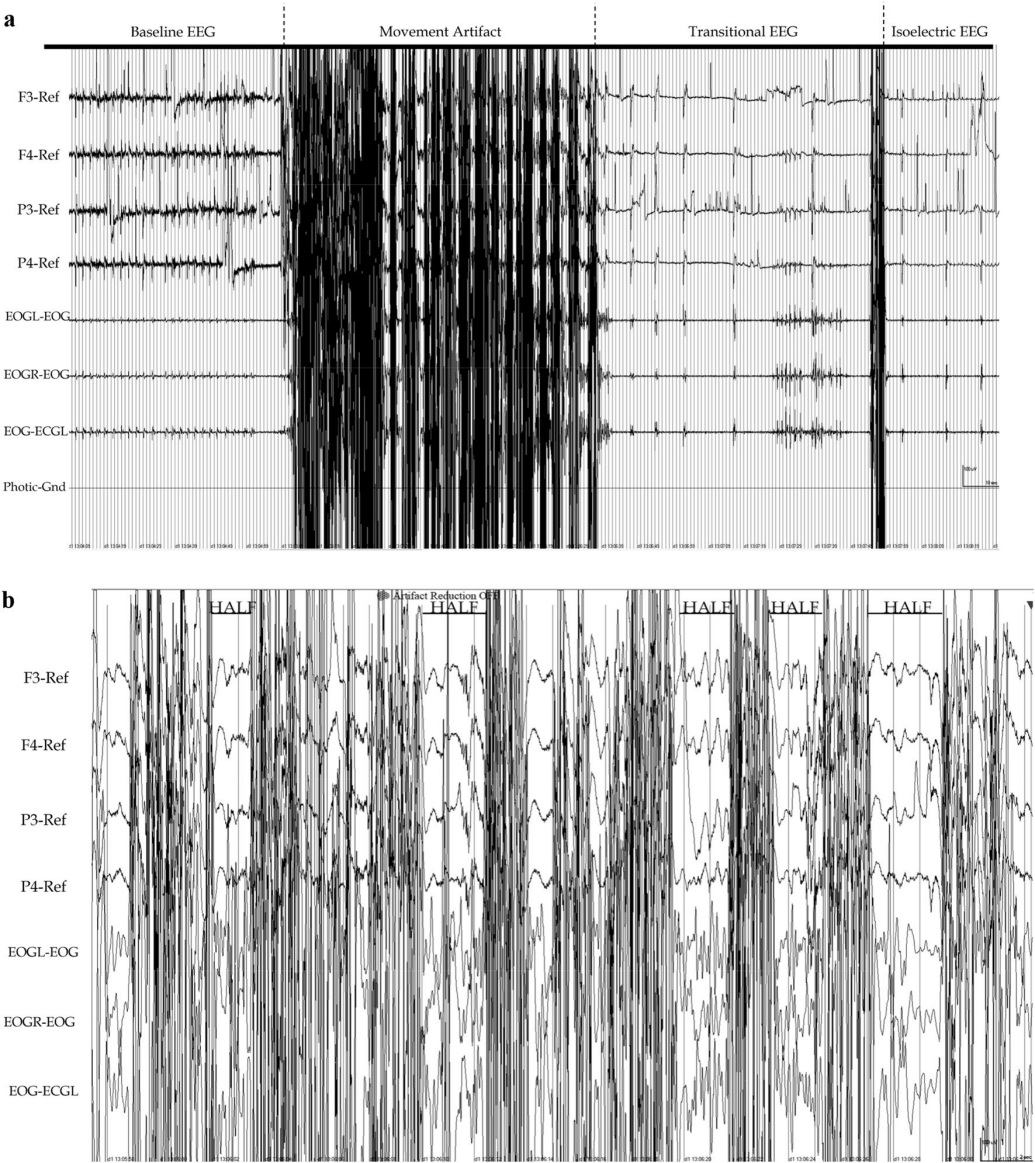
(**a**) Compressed EEG (4 min, 20 s) on one of the pigs, demonstrating baseline EEG before intervention (“Baseline EEG”), movement artifact, transitional and isoelectric EEG. Movement artifact showed a continuous and intermittent component. The EEG became transitional immediately after movement stopped and shifted rapidly to an isoelectric pattern. High pass filter: 0.1, Low pass filter: 15 Hz. (**b**) EEG showing high amplitude low frequency (HALF) EEG activity observed during the intermittent component of movement artifact in all pigs. High pass filter: 0.1, Low pass filter: 15 Hz. (**a**) and (**b**) were created using Persyst software version 13 rev. D (Persyst, Solana Beach, CA, USA; https://www.persyst.com/).

Collected EEG data was interpreted by Dr. Vidaurre, a specialist in clinical neurophysiology and EEG pattern analysis, using Persyst software version 13 rev. D (Persyst, Solana Beach, CA, USA) and following the EEG pattern classification system described by Gibson et al.^25^. In brief, the classified patterns were normal EEG (baseline), transitional EEG, high amplitude low frequency (HALF) EEG, isoelectric EEG and movement artifacts. Baseline EEG was captured for five minutes prior to foaming. Transitional EEG was defined as EEG with amplitude of less than half of that of baseline EEG, and HALF EEG was defined as waveforms of high amplitude with low frequency activity. Isoelectric EEG was defined as electrical activity with amplitude of < 1/8 (12.25%) of that of baseline EEG, or EEG with little or no identifiable brain activity^25^. Lastly, movement artifacts were defined as any electrical activity non-representative of brain-derived waveforms^26^, caused by muscular activity during the foaming process. An example of EEG patterns as defined here is shown in Fig. 2. EEG data collection continued for 15 min post foam fill of the container.

Both transitional and HALF EEG were designated as unconscious states as previously described^25,27^, where transitional EEG is a conservative estimate of unconsciousness and HALF EEG demonstrates characteristics of unconsciouness. Brain death was determined at the onset of the isoelectric pattern (Fig. 2a). Data was manually imported to Excel (Microsoft, Redmond, CA) and descriptive statistical analysis was conducted to describe the time to unconsciousness and death (mean ± SD) for each individual animal, and then as summaries for the entire experimental cohort. Further statistical models were not built considering the limited sample size, the intentional selection of subjects, and the nature of the primary objective, and results are presented primarily descriptively. The datasets used and/or analyzed during the current study are available from the corresponding author on reasonable request.

### Ethics approval

All methods utilized in this study were performed in accordance with the relevant guidelines and regulations, including approval by The Ohio State University Institutional Animal Care and Use Committee (protocol 2020A00000036) and the Guide for the Care and Use of Agricultural Animals in Research and Teaching.

## Results

All 12 foaming events resulted in successful depopulation by post WBF assessment and confirmation of death as previously described. Time to fill the container with foam was between 3 and 4 s for all replicates. The average time to end of movement artifact was 1 min, 49 s ± 33 s (max = 2 min, 57 s, min = 1 min, 16 s), with HALF EEG representing characteristics of unconsciousness observed in the intermittent component of movement artifact for each pig. Movement artifacts were followed by transitional EEG immediately after cessation of movement artifact for most animals. Exceptions included subject 9, which showed transitional EEG before main movement artifact stopped; subject 10, which showed brief normal EEG after movement artifacts, then transitional EEG, followed by isoelectric EEG. Lastly, subject 3 showed transitional EEG right after movement artifacts, then normal EEG, transitional EEG, and finally isoelectric EEG. The average time from WBF immersion to final transitional EEG (i.e., unconsciousness) was 1 min, 53 s ± 36 s (max = 2 min, 57 s, min = 1 min 16 s). The average duration of transitional EEG was 1 min, 14 s ± 42 s (max = 2 min, 19 s, min = 19 s). The average time from WBF immersion to isoelectric EEG (i.e., brain death) was 3 min, 3 s ± 56 s (max = 4 min, 52 s s, min = 1 min, 36 s). Individual animal’s time in each EEG phase are identified in Table 1.

**Table 1:** Objective data from each animal including sex, weight, and time in each EEG phase with averages in the bottom row.

Subject	Sex	Weight (kg)	Delay in onset of movement (m:ss)	Movement artifact (m:ss)	Intermittent transitional EEG (m:ss )	Intermittent normal EEG (m:ss)	Transitional EEG (m:ss)	Isoelectric EEG (m:ss)
Pig 1	F	15.47	0:12	2:38			0:51	11:35
Pig 2	F	13.29	0:10	1:11			1:15	12:16
Pig 3	F	15.46	0:37	1:03	0:46	0:10	0:28	8:42
Pig 4	F	13.40	0:12	1:24			0:28	10:17
Pig 5	F	12.91	0:00	1:16			2:19	6:57
Pig 6	F	11.65	0:17	1:22			2:14	11:04
Pig 7	M	15.01	0:08	1:56			1:41	8:16
Pig 8	M	13.04	0:05	1:12			0:19	13:49
Pig 9	M	13.39	0:00	1:52			0:38	9:50
Pig 10	M	12.91	0:00	1:29		0:01	1:26	7:24
Pig 11	M	13.10	0:02	2:55			1:55	8:04
Pig 12	M	12.93	0:24	1:18			0:27	10:06
Average		13.55	0:11	1:38			1:10	9:52

## Discussion

In nursery pigs, WBF consistently induced unconsciousness, which quickly progressed to irreversible brain death. The observed average time of 3 min, 3 s from WBF immersion to brain death correspond well with observations by Williams et al.^28^, where a dwell time between 2 min, 30 s and 5 min of WBF immersion was required to induce a non-recoverable state. The EEG results in the present study provide support to the 7 min, 30 s dwell time recommendation for depopulation using WBF by Williams et al.^28^. The 7 min, 30 s recommendation would provide possible buffer time to account for any outliers as observed in Pig 11, who did not reach an isoelectric state until 4 min, 52 s post WBF immersion.

Consciousness, in general, poses some of the “most baffling problems” in the studies of the brain^29^. Determination of consciousness is especially problematic in the evaluation of WBF as a depopulation approach because WBF occludes the ability to actively monitor insensibility in the pig. The use of EEG data as an indicator of loss of consciousness and insensibility in animals has been applied during anesthesia^30^, stunning, and euthanasia^21,23,25,27,31–33^. Thus, EEG has been regarded as one of the most objective approaches to evaluate consciousness^27,34^, although several definitions of brain activity phases have been used in the context of animal depopulation^23,25,27^.

The conservatively measured 1 min, 53 s average time to unconsciousness based on EEG analysis in the present study was shorter than the 2 min, 28 s required for cessation of movement (COM) reported by Lorbach et al.^12^, in which COM was used as a metric to approximate unconsciousness. The difference between EEG and COM results can likely be attributed to agonal movements occurring after the loss of consciousness, thus making the COM an overestimate of time to induce loss of consciousness. The differences in estimates highlight the urgent need for validated and standardized assessments of loss of consciousness/insensibility and death parameters for depopulation studies.

Comparisons between EEG studies are currently hampered by differences among species, variation among individuals, differences in equipment, inability to pinpoint exact time of unconsciousness, and placement of electrodes^27^. The present study was restricted to one age class and weight range (i.e., nursery pigs) given concerns of the possible interference of EEG measurement in larger animals due to limitations on immobilizing them appropriately. Additionally, per previously established definitions^25^, transitional EEG is a conservative measure of unconsciousness, while HALF EEG is representative of characteristics of unconsciousness; however, it is difficult to record an exact moment when HALF EEG occurs due to this phase being buried in the intermittent component of movement artifact, which makes it difficult to assess precise times to unconsciousness. Furthermore, subdermal electrodes have been associated with noises on EEG interpretation due to the skull’s low electrical conductivity^35^, which could be reduced using an ‘under the skull’ epidural EEG^21^. However, the latter approach was not used because there are increased risks associated with the ‘under the skull’ approach which can lead to unwanted mortality^21^. It is also important to note that the way animals were restrained (using a sling) could have impacted time to unconsciousness and brain death; which is important because this is not reflective of how animals would be depopulated under field conditions. We hypothesize that, without immobilization, animals could have had decreased time to loss of consciousness and more rapid brain death due to an expected increase in respiration rate due to freedom of movement and possible faster oxygen deprivation.

The use of WBF for swine depopulation has not been well explored^36^; however, the use of WBF as a depopulation method for floor-reared poultry is well documented^37–41^. Alphin et al.^29^ depopulated 68 broilers in four groups with either Argon-CO_2_, CO_2_ gas, WBF with ambient air, and WBF with CO_2_. While Argon-CO_2_ took the longest time to induce brain death, there was no statistical difference when comparing CO_2_ gas, WBF with ambient air, and WBF with CO_2_. However, the study did not try to estimate the time for birds to reach unconsciousness. In a study by Rankin et al.^40^, latency to brain death was shorter for turkeys depopulated with WBF when compared with CO_2_. However, while the latency to unconsciousness was numerically shorter for WBF when compared with CO_2_, the difference was not statistically significant.

This study also had limitations. As previously noted, the limited sample size and restriction of subjects to healthy animals of a particular size and age may reduce generalizability of study results. Additionally, the immobilization of animals by the use of the sling coupled with the obstructive visual nature of the WBF method limited assessment of other important welfare-related behaviors, such as escape attempts, breathlessness, and gasping, which did not allow for conclusions regarding method aversiveness, an important consideration when considering animal welfare for these emergency depopulation methods. Lastly, having a group of animals that would had been depopulated using AVMA- approved methods (e.g. CO_2_) would had been beneficial to allow for comparison across different types of depopulation methods. However, reduction of number of individuals is a priority for terminal studies, and the primary study goal was to investigate a new depopulation approach which is currently not an AVMA approved method, foam; and not to make comparison across depopulation methods. Further research should focus on using EEG to investigate time to loss of consciousness with various depopulation methods in swine of all ages, and the addition of comparison groups using other AVMA-approved depopulation methods. In summary, results of the present study indicate that the use of WBF was efficient in causing loss of consciousness followed by death. Other advantages of using WBF for swine depopulation include ease of implementation, cost effectiveness, and the potential for improved mental health and safety implications for personnel.

## Conclusion

The EEG findings of the present study provide additional scientific information on time to loss of consciousness and brain death in nursery pigs exposed to WBF depopulation. The average (SD) 1 min, 53 s ± 36 s for time to loss of consciousness supports previously published research characterizing WBF and confirms that WBF is an effective and suitable candidate for swine depopulation. While there is no perfect depopulation method for every situation, WBF has the potential to meet the U.S. swine industry’s need for a depopulation strategy that could be applied to pigs in all stages of production, applicable across various production systems, and quickly implemented in case of outbreaks of foreign animal disease or emergency events.

## Data Availability

The datasets used and/or analyzed during the current study available from the corresponding author on reasonable request.
